# Evaluating the role of selection in the evolution of mitochondrial genomes of aboriginal peoples of Siberia

**DOI:** 10.18699/VJGB-23-28

**Published:** 2023-06

**Authors:** B.A. Malyarchuk, M.V. Derenko

**Affiliations:** Institute of Biological Problems of the North of the Far Eastern Branch of the Russian Academy of Sciences, Magadan, Russia; Institute of Biological Problems of the North of the Far Eastern Branch of the Russian Academy of Sciences, Magadan, Russia

**Keywords:** mitochondrial DNA, natural selection, Ka/Ks-testing, human populations, Siberia, митохондриальная ДНК, естественный отбор, Ka/Ks-тесты, популяции человека, Сибирь

## Abstract

Studies of the nature of mitochondrial DNA (mtDNA) variability in human populations have shown that protein-coding genes are under negative (purifying) selection, since their mutation spectra are characterized by a pronounced predominance of synonymous substitutions over non-synonymous ones (Ka/Ks < 1). Meanwhile, a number of studies have shown that the adaptation of populations to various environmental conditions may be accompanied by a relaxation of negative selection in some mtDNA genes. For example, it was previously found that in Arctic populations, negative selection is relaxed in the mitochondrial ATP6 gene, which encodes one of the subunits of ATP synthase. In this work, we performed a Ka/Ks analysis of mitochondrial genes in large samples of three regional population groups in Eurasia: Siberia (N = 803), Western Asia/Transcaucasia (N = 753), and Eastern Europe (N = 707). The main goal of this work is to search for traces of adaptive evolution in the mtDNA genes of aboriginal peoples of Siberia represented by populations of the north (Koryaks, Evens) and the south of Siberia and the adjacent territory of Northeast China (Buryats, Barghuts, Khamnigans). Using standard Ka/Ks analysis, it was found that all mtDNA genes in all studied regional population groups are subject to negative selection. The highest Ka/Ks values in different regional samples were found in almost the same set of genes encoding subunits of ATP synthase (ATP6, ATP8), NADH dehydrogenase complex (ND1, ND2, ND3), and cytochrome bc1 complex (CYB). The highest Ka/Ks value, indicating a relaxation of negative selection, was found in the ATP6 gene in the Siberian group. The results of the analysis performed using the FUBAR method (HyPhy software package) and aimed at searching for mtDNA codons under the influence of selection also showed the predominance of negative selection over positive selection in all population groups. In Siberian populations, nucleotide sites that are under positive selection and associated with mtDNA haplogroups were registered not in the north (which is expected under the assumption of adaptive evolution of mtDNA), but in the south of Siberia.

## Abstract (ru)

Исследования характера изменчивости митохондриальной ДНК (мтДНК) в популяциях человека выявили, что белок-кодирующие гены находятся под действием отрицательного (очищающего) отбора, поскольку мутационные спектры генов мтДНК характеризуются выраженным преобладанием синонимичных замен над несинонимичными (величина параметра Ka/Ks < 1). Между тем в ряде исследований показано, что адаптация популяций к различным условиям природной среды может сопровождаться ослаблением отрицательного отбора в некоторых генах мтДНК. Так, ранее было установлено, что в арктических популяциях отрицательный отбор ослаблен в митохондриальном гене ATP6, кодирующем одну из субъединиц АТФ-синтазы. В настоящей работе проведен Ka/Ks-анализ митохондриальных генов в больших выборках трех региональных групп населения Евразии: Сибири (N = 803), Западной Азии/Закавказья (N = 753) и Восточной Европы (N = 707). Основная цель работы – поиск следов адаптивной эволюции в генах мтДНК коренного населения Сибири, представленного населением севера (коряки, эвены) и юга Сибири и прилегающей территории Северо-Восточного Китая (буряты, баргуты, хамнигане). С помощью стандартного Ka/Ks-анализа установлено, что все гены мтДНК во всех изученных региональных группах населения испытывают действие отрицательного отбора. Наиболее высокие значения Ka/Ks в различных региональных выборках обнаружены практически в одном и том же наборе генов, кодирующих субъединицы АТФ-синтазы (ATP6, ATP8), НАДН-дегидрогеназного комплекса (ND1, ND2, ND3) и цитохром bc1- комплекса (CYB). Самое высокое значение Ka/Ks, указывающее на ослабление отрицательного отбора, выявлено в гене ATP6 в сибирской группе. Результаты анализа, выполненного с помощью метода FUBAR (пакет программ HyPhy) и направленного на поиск кодонов мтДНК, находящихся под действием отбора, также показали преобладание влияния отрицательного отбора над положительным отбором во всех группах населения. В сибирских популяциях нуклеотидные позиции, находящиеcя под действием положительного отбора и ассоциированные с гаплогруппами мтДНК, зарегистрированы не на севере (что ожидается в предположении адаптивной эволюции мтДНК), а на юге Сибири.

## Introduction

Mitochondrial DNA (mtDNA) is a valuable tool for studying the evolutionary history of humans, which is associated with such features of the mitochondrial genome as maternal inheritance without recombinations and a high mutation rate compared to the nuclear genome (Brown et al., 1979; Giles et al., 1980). The gradual accumulation of mutations in mtDNA haplotypes leads to the formation of groups of phylogenetically related haplotypes (i. e., mtDNA haplogroups), which are characterized by a population-specific distribution (Wallace, 1995). At the beginning of studies, continental macrohaplogroups were discovered, and later, as the resolution of mtDNA analysis increased – from sequencing of certain mtDNA regions to sequencing of complete mitogenomes – ethnospecific mtDNA haplogroups were identified (Olivieri et al., 2017; Derenko et al., 2019; García et al., 2020).

The results of population genetic studies of the last 20 years point to the great importance of negative (purifying) selection in human mitochondrial genome evolution (Mishmar et al., 2003; Elson et al., 2004; Kivisild et al., 2006; Ingman, Gyllensten, 2007; Sun et al., 2007; Derenko, Malyarchuk, 2010; Eltsov et al., 2010; Malyarchuk, 2011; Litvinov et al., 2020). This is primarily due to significance of this genetic system, which ensures the effective functioning of the mitochondrial respiratory chain. Genes encoding subunits of protein complexes of the respiratory chain (NADH-ubiquinone-oxidoreductase, cytochrome bc1, cytochrome c-oxidase, ATP-synthase) make up about 70 % of the mitochondrial genome. The high stability of these genes is due to the significant prevalence of synonymous substitutions in various mtDNA genes (Ks) over non-synonymous ones (Ka), leading to amino acid substitutions.

Meanwhile, early studies have shown that mitochondrial genes may be subject to positive selection, leading to the prevalence of non-synonymous substitutions over synonymous ones, due to the adaptation of human populations to various natural environmental conditions (Mishmar et al., 2003). Thus, a deviation from the neutral model of mtDNA variability was found in different population groups of Eurasia and America. An analysis of the distribution of Ka/Ks values has shown that negative selection is relaxed in the mitochondrial ATP6 gene in the Arctic zone, in the CYB gene in the temperate zone (Europe), and in the CO1 and ND3 genes in the tropics (Mishmar et al., 2003; Ingman, Gyllensten, 2007).

The prevalence of elevated Ka/Ks values in the ATP6 gene in the Arctic zone (in Siberian and North American populations) in comparison with other regions was explained by the adaptation of populations to the Far North environmental conditions (Mishmar et al., 2003). The ATP6 gene encodes subunit 6 of mitochondrial ATP synthase, which is involved in the coupling of ATP production and heat to maintain body temperature, and therefore it is suggested that polymorphic variants that reduce coupling efficiency may be beneficial under cold stress conditions, as they increase heat production and overall metabolic rate (Mishmar et al., 2003).

Later, it was also shown that higher Ka/Ks values in the ATP6 gene prevail in East Asians (Elson et al., 2004; Sun et al., 2007). In another study of the mitochondrial genomes of the North Asian populations, the highest Ka/Ks values were also found in the ATP6 gene (Ingman, Gyllensten, 2007). The accumulation of non-synonymous mutations in this gene was considered by the authors as an evolutionarily slow process of gradual relaxation of negative selection over many thousands of years. This scenario is also supported by evidence that some ancient non-synonymous substitutions were defining mtDNA haplogroups that have become widespread in northern Asia (Ruiz-Pesini et al., 2004). For example, there is the G8584A substitution, which defines the M8 macrohaplogroup and its predominantly North Asian haplogroup C, the C8794T substitution, which determines the haplogroup A, and the A8701G substitution, which delineates the macrohaplogroup N as a whole. It is assumed that such mtDNA replacements are associated with changes in energy metabolism and, thus, contributed to adaptation to northern conditions, being potential candidates for adaptive selection (Ruiz-Pesini et al., 2004).

Another scenario, as noted above, is that mutational changes in the ATP6 gene occurred as a result of relaxed negative selection, and the increase in the frequency of these polymorphic variants in northern populations was facilitated by genetic drift, the effects of which are better manifested in populations with a small effective size (Ingman, Gyllensten, 2007).

The results of Ka/Ks analysis of mitochondrial genes in cancer tissues demonstrated a significant relaxation of negative selection in many mtDNA genes under conditions of aerobic glycolysis, which actively occurs in cancer cells (Stafford, Chen-Quin, 2010; Liu et al., 2012; Skonieczna et al., 2018). When comparing the Ka/Ks spectra in healthy and cancerous tissues, it turned out that in the mitochondrial genes of affected cells in various types of cancer, a statistically significant relaxation of negative selection is observed in all genes, except for ATP6 and ATP8, as well as ND3 and CO2 (Liu et al., 2012). With respect to the genes encoding subunits of ATP synthase, this means that the mitochondria of healthy cells are likely to be characterized by such a significant relaxation of negative selection that it practically does not differ from that under conditions of carcinogenesis. However, the reasons for such behavior of the mitochondrial ATP6 and ATP8 genes in the norm are not fully understood.

Thus, the results of studies of the evolution of proteincoding mtDNA genes in human populations testify to a rather high stability of mitochondrial genes; however, the differences that were revealed between populations indicate a possible influence of positive selection on some mtDNA genes due to the adaptation of populations to different climatic conditions. In a number of studies, this issue was considered based on populations of East Asia, including the aboriginal populations of Siberia, but the sample sizes of populations studied were insufficiently representative (less than 100 complete mitogenomes) (Mishmar et al., 2003; Elson et al., 2004; Kivisild et al., 2006; Ingman, Gyllensten, 2007; Sun et al., 2007).

In this paper, we present more detailed information on the effect of selection on the mitochondrial genomes of human populations based on the results of Ka/Ks analysis of mtDNA genes in aboriginal populations of Siberia (N = 803) in comparison with populations of Western Asia and Transcaucasia (N = 753) and Eastern Europe (N = 707).

## Materials and methods

Whole mtDNA genome data from Siberian and East Asian populations published in GenBank (https://www.ncbi.nlm.nih. gov/genbank/) were analyzed. The data are represented by the Koryaks (N = 154) and Evens (N = 219) from the northern part of Siberia, and by the Mongolic-speaking Buryats, Barghuts and Khamnigans (N = 430) from the southern part of Siberia and adjacent territories of Northeast China. For comparison, we used data on the whole mitogenome variability in populations of Western Asia (Persians, Qashqais, Lebanese) and Transcaucasia (Armenians and Azeri) (N = 753), as well as in populations of Eastern Europe (Russians, Ukrainians, Volga Tatars and Estonians) (N = 707).

We analyzed the distribution of Ka/Ks values (the ratio of the number of non-synonymous substitutions for a nonsynonymous site (Ka) to the number of synonymous substitutions for a synonymous site (Ks)) in the mtDNA L- strand encoded genes ND1, ND2, CO1, CO2, ATP8, ATP6, CO3, ND3, ND4L, ND4, ND5 and CYB. For Ka/Ks analysis, we used the programs of the package DnaSP v. 5 (Librado, Rozas, 2009). The effect of negative selection is assumed at Ka/Ks <1 and positive selection at Ka/Ks > 1. To analyze the effect of selection on mtDNA protein-coding genes, the HyPhy software package was also used (http://www.hyphy.org) (Kosakovsky Pond et al., 2005). To identify codons under the influence of negative and positive selection, the FUBAR method (Fast Unconstrained Bayesian AppRoximation) was used. This method allows you to quickly analyze large sets of molecular data using the hierarchical Bayesian method and the Monte Carlo method for Markov chains (MCMC) (Murrel et al., 2013).

## Results and discussion

The results of the analysis of the distribution of Ka/Ks values in the protein-coding genes of the mitochondrial genome in aboriginal populations of Siberia demonstrate that in all but one of the cases, the values of this parameter are below 1, which indicates the effect of negative selection on mtDNA genes (Table 1). The highest Ka/Ks values were detected in the ATP6 gene. Moreover, among the Koryaks, a Paleo-Asiatic people that originated in Northeast Asia under extreme environmental conditions, the Ka/Ks value exceeds 1, which indicates the effect of positive selection on this mitochondrial gene

**Table 1.**
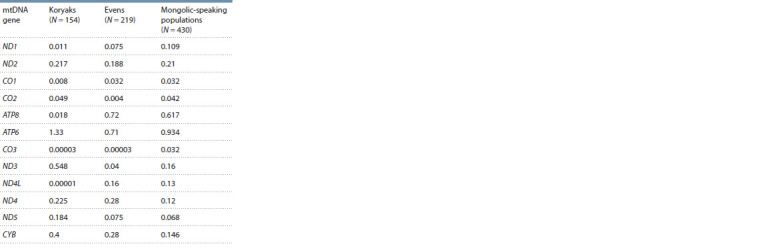
Ka/Ks values for mtDNA genes in aboriginal populations of Siberia

Table 2 shows Ka/Ks values in three regional population groups. In the Siberian group of populations, the highest values were found in the ATP6, ATP8, ND2, and CYB genes; in the populations of Western Asia and Transcaucasia – in the ATP6, ATP8, ND1, ND2, and CYB genes; in the populations of Eastern Europe – in the ATP6, ATP8, ND1, ND3, and CYB genes. Therefore, the results of analysis indicate that in different regions of Eurasia the highest Ka/Ks values are found in about the same sets of mitochondrial genes. The maximum values of this parameter were revealed in the genes encoding subunits of ATP synthase, which is consistent with the results of previous studies (Mishmar et al., 2003; Ingman, Gyllensten, 2007; Sun et al., 2007) and points to a relaxation of negative selection in the ATP6 and ATP8 genes, especially in Siberian populations.

**Table 2.**
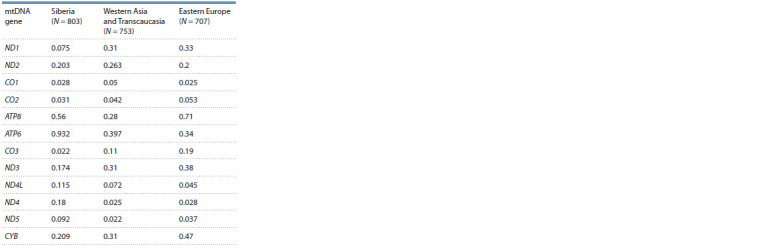
Ka/Ks values for mtDNA genes in regional populations

To assess selective pressure acting on individual mtDNA sites (with taking into account their location in the phylogenetic tree of mtDNA haplotypes), we used hierarchical Bayesian analysis implemented in the FUBAR program of the HyPhy package (http://www.hyphy.org). This method has a higher efficiency of detecting codons that are under the influence of positive and negative selection – for example, in comparison with the FEL (Fixed Effects Likelihood) and MEME (Mixed Effects Model of Evolution) methods of the HyPhy package, which are also widely used to study selective processes (Murrel et al., 2012, 2013).

Our study demonstrated that in Siberian populations, 11.4 % (411) codons, which are roughly evenly distributed over the mtDNA genes, are under the influence of negative selection. The effect of positive selection was found only in 4 codons of the ND5 and CYB genes (Table 3).

**Table 3.**
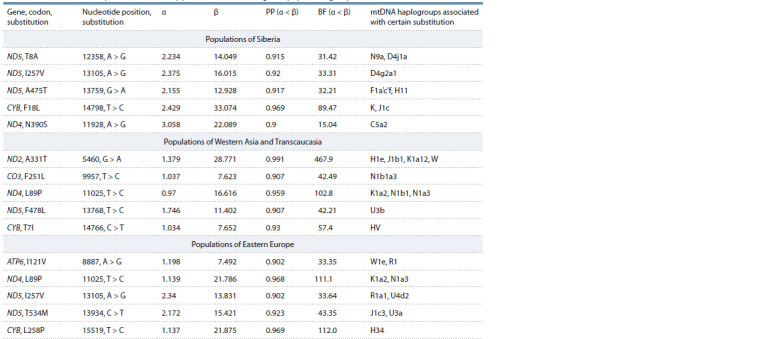
mtDNA nucleotide positions affected by positive selection in regional population groups (FUBAR method) Notе. α – rate of synonymous substitutions; β – rate of non-synonymous substitutions; PP – posterior probability; BF – Bayes factor. PP for codons, which are under the influence of positive selection, is > 0.9.

When only the populations of the northeastern part of Siberia (Koryaks and Evens) are analyzed, another codon, which is characterized by a borderline posterior probability value of 0.9, is revealed in the ND4 gene (see Table 3). In this case, the nucleotide substitution in the ND4 gene, leading to the N390S amino acid substitution, determines the C5a2 phylogenetic cluster, which is interesting in that it is distributed mainly among the Koryaks.

All other substitutions are found in mtDNA clusters characterized by a more southern distribution – they are associated either with major mtDNA haplogroups widespread in East and South Asia (for example, N9a and F1a’c’f) or in Western Eurasia (for example, H11, K, J1c), or with relatively small East Asian mtDNA haplogroups found also in Buryats, Barghuts, and Khamnigans (D4j1a, D4g2a1) (see Table 3).

Thus, oddly enough, despite the expected effect of positive selection on individual mtDNA sites due to the adaptation of aboriginal populations of Northeastern Siberia to a cold climate, the effect of positive selection was found only in southernmost Siberian populations. Similar conclusions follow from the results of the analysis of mtDNA protein-coding genes in Siberian populations obtained using other methods, FEL and MEME (results not shown).

For comparison, data sets for populations of Western Asia/ Transcaucasia and Eastern Europe were also analyzed (see Table 3). In the first case, 19.5 % (700) codons were found under the influence of negative selection, in the second case – 16.4 % (589) codons. Under the influence of positive selection, five codons were identified in both regional population groups (see Table 3). All substitutions are associated with mtDNA haplogroups widespread in Western Eurasia, and therefore it is difficult to accept (at least in the absence of a special analysis) that the fixation of these substitutions in haplogroup trunks occurred due to the adaptation of populations to natural environmental conditions. It should be noted that in two cases there is evidence of the influence of positive selection on the same codon in different geographic regions: a nucleotide sub-stitution at position 11025, which determines the haplogroups K1a2 and N1a3, in the populations of Western Asia/ Transcaucasia and Eastern Europe, and a nucleotide substitution at position 13105, which defines haplogroup D4g2a1 in Siberian populations and haplogroups R1a1 and U4d2 in populations of Eastern Europe (see Table 3).

## Conclusion

Thus, our study aimed at the effects of selection on mtDNA genes in different regional groups of Eurasia using standard Ka/Ks analysis showed that all mtDNA genes are characterized by low values of this parameter (Ka/Ks < 1), indicating the influence of negative selection. The highest Ka/Ks values in different regional population groups were found in almost the same set of genes encoding subunits of ATP synthase (ATP6, ATP8), NADH dehydrogenase complex (ND1, ND2, ND3), and cytochrome bc1 complex (CYB). The highest value of Ka/Ks, indicating a relaxation of negative selection, was found in the ATP6 gene in Siberian populations; moreover, in Koryaks, the effect of positive selection on this gene was formally recorded (Ka/Ks = 1.33).

Meanwhile, the results of the analysis aimed at searching for mtDNA codons affected by selection showed a multiple prevailing negative selection over positive one in all population groups under study. In Siberian populations, codons affected by positive selection and associated with mtDNA haplogroups have been revealed only in populations of the southern part of Siberia and the adjacent territory of Northeast China (among the Buryats, Barghuts, and Khamnigans). In the regional groups of Eurasian populations, codons of this kind were found in different mtDNA genes (ND2, ND4, ND5, CO3, CYB), but in the ATP6 gene a single codon (at position 121) was detected in the East European group of populations rather than in the Siberian one. Apparently, further studies of the direction and strength of natural selection on mitochondrial genomes in different regional population groups of Eurasia are required

## Conflict of interest

The authors declare no conflict of interest.
